# Limiting Wind-Induced Resuspension of Radioactively Contaminated Particles to Enhance First Responder, Early Phase Worker and Public Safety—Part 1

**DOI:** 10.3390/app12052463

**Published:** 2022-02-26

**Authors:** Hadas Raveh-Amit, Avi Sharon, Itzhak Katra, Terry Stilman, Shannon Serre, John Archer, Matthew Magnuson

**Affiliations:** 1 Department of Chemistry, Nuclear Research Centre Negev, P.O. Box 9001, Beer Sheva 8419000, Israel; 2 Environmental Research Unit, Nuclear Research Centre Negev, P.O. Box 9001, Beer Sheva 8419000, Israel; 3 Department of Geography and Environmental Development, Ben-Gurion University, P.O. Box 653, Beer Sheva 8410501, Israel; 4 EPA Region 4, Atlanta, GA 30303, USA; 5 EPA Office of Land and Emergency Management, Washington, DC 20002, USA; 6 EPA Office of Research and Development, Cincinnati, OH 45268, USA;

**Keywords:** soil resuspension, stabilization material, soil contamination, Dead Sea saltwater, dust emission, soil erosion

## Abstract

An accidental radiological release or the operation of a radiological dispersal device (RDD) may lead to the contamination of a large area. Such scenarios may lead to health and safety risks associated with the resuspension of contaminated particles due to aeolian (wind-induced) soil erosion and tracking activities. Stabilization technologies limiting resuspension are therefore needed to avoid spreading contamination and to reduce exposures to first responders and decontamination workers. Resuspension testing was performed on soils from two sites of the Negev Desert following treatment with three different stabilization materials: calcium chloride, magnesium chloride, and saltwater from the Dead Sea in Israel. Two and six weeks post-treatment, resuspension was examined by inducing wind-driven resuspension and quantitatively measuring particle emission from the soils using a boundary-layer wind tunnel system. Experiments were conducted under typical wind velocities of this region. Treating the soils reduced resuspension fluxes of particulate matter < 10 μm (${PM}_{10}$) and saltating (sand-sized) particles to around background levels. Resuspension suppression efficiencies from the treated soils were a minimum of 94% for all three stabilizers, and the Dead Sea salt solution yielded 100% efficiency over all wind velocities tested. The impact of the salt solutions (brine) was directly related to the salt treatment rather than the wetting of the soils. Stabilization was still observed six weeks post-treatment, supporting that this technique can effectively limit resuspension for a prolonged duration, allowing sufficient time for decision making and management of further actions.

## 1. Introduction

An accidental radiological release or the operation of a Radiological Dispersal Device (RDD) may lead to the contamination of a large area with radioactive materials. During the immediate emergency phase of a response, life-saving operations and securing of critical infrastructure must be conducted for the safety of the public and first responders [1,2]. During the operations, emergency responders, as well as decontamination workers assisting with the response, may be further exposed due to inhalation of resuspended particles and direct contact, owing to the tracking of contamination from the contaminated areas, i.e., roads, other construction materials and soils. Containment of the contaminated area to prevent resuspension could reduce the overall exposure for emergency responders and decontamination workers and also reduce the spread of contamination. Hence, stabilization technologies and methodologies to minimize this exposure are needed [1,2].

Aeolian (wind-induced) soil erosion, and the following process of dust emission, results in the resuspension of soil-derived particles to the atmosphere and air pollution [3–5]. Stabilization technologies are designed to prevent the spread of particles (such as by resuspension) and are routinely used in industries, such as road constructions and mining sites, for dust control [2]. The application of rapidly available and easily applied stabilization technologies has the potential for accomplishing multiple goals following the release of radioactive particles from a radiological contamination event. Primarily, the application of a stabilization material may reduce exposures to first responders and decontamination workers assisting with the response due to tracking. In addition, such technologies would limit the wind-induced spread of contamination to other non-contaminated, less-contaminated, or recently decontaminated areas, subsequently reducing the time and resources needed for additional decontamination operations [2].

The United States Environmental Protection Agency (EPA) previously conducted work on stabilization technologies [1,2,6,7]. From these studies, the list below presents some options recommended by stakeholders and experts that may be suitable for stabilization:

- Soil2O^®^1 dust control wetting agent (available in the US);
- Calcium chloride (CaCl_2_);
- Phos-Chek^®^MVP-F3 fire retardant (available in the US);
- Locally available firefighting foam;
- Capping with locally available gravel, mulch, sand or clay;
- Misting with water or saltwater (brine), with the possible addition of additives;
- Application of a polymer coating/gel.

There is a lack of fundamental research examining the applicability of stabilization materials required in an event leading to the contamination of a large area with radioactive materials. Stabilization materials suitable for large areas of contaminated soils are expected to be cheap, easily applied and highly effective in limiting wind-induced contamination dispersal. Previous works showed the potential of specific brines to reduce dust emission from unpaved roads of different soils [3,8,9], with low environmental salinization risk [10]. The current study aimed to test the effectiveness of different brines to stabilize arid soils that may be subjected to soil contamination and are already associated with natural dust emission.

## 2. Materials and Methods

### 2.1. Soil Sampling and Physicochemical Characterization

Soils were sampled from two sites that are undisturbed and associated with dust emission in the field: Ze’elim sandy area (31.16° E/34.53° N) at the western Negev Desert [11], and the Yamin plateau (31.04° E/35.08° N) at the northeastern Negev Desert in Israel [12]. The soil samples were analyzed for elemental composition by X-ray fluorescence (XRF) using an Axios spectrometer (PANanalytical, Malvern, UK). Mineralogical phase identification was performed by X-ray powder diffraction (XRPD) using an Empyrean Philips 1050/70 diffractometer (PANanalytical, Malvern, UK). Particle size distribution (PSD) was performed by laser diffraction using Analysette 22 MicroTec Plus (Fritsch International, Idar-Oberstein, Germany). XRF, XRPD and PSD analyses were performed at the Ben-Gurion University of the Negev in Beer-Sheva. pH was measured using a Metrohm pH meter (Metrohm, Herisau, Switzerland). Water content in soils was measured gravimetrically. Total organic content (TOC) was determined by titration of the dissolved organics with ammonium iron sulfate using an 848 Titrino plus (Metrohm, Herisau, Switzerland) at the Geological Survey of Israel.

### 2.2. Soil Stabilization

#### 2.2.1. Stabilization Materials

**Figure 1.**
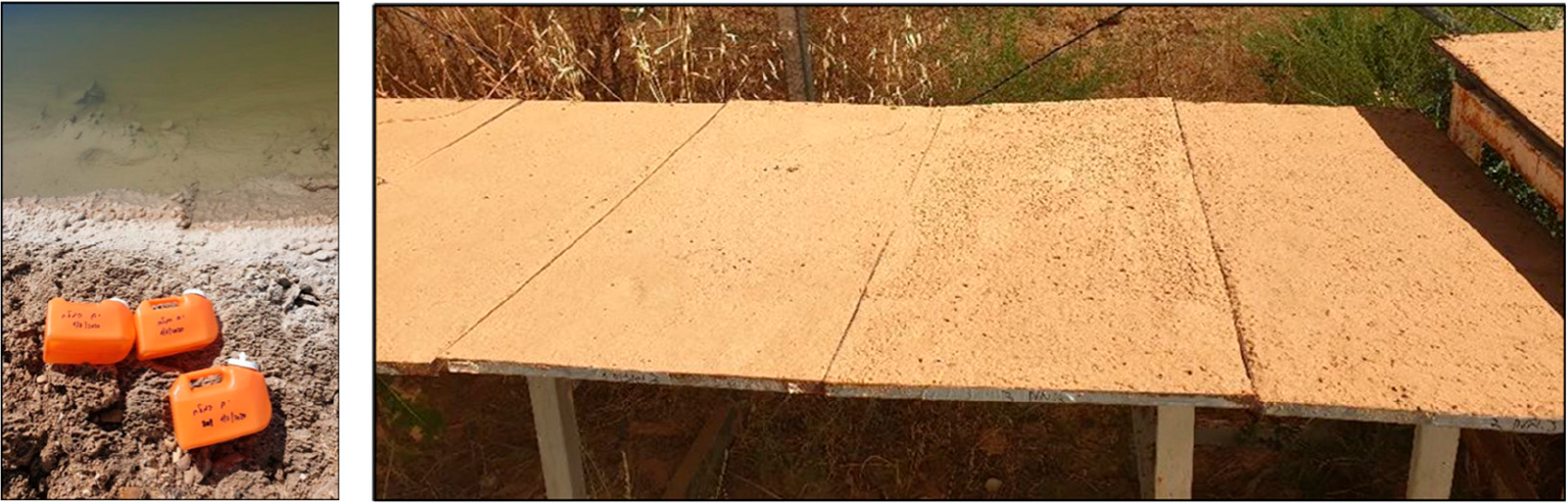
Samples of Dead Sea salt solutions collected in 3 L containers (left side) and trays of Ze’elim soil treated with different brines (surface area of 0.5 m × 1.0 m and height of 0.02 m).

Three brine solutions were tested in this study: magnesium chloride (MgCl_2_), calcium chloride (CaCl_2_) and a solution sampled from the Dead Sea in Israel. MgCl_2_ and CaCl_2_ salts were purchased from Carlo Erba Reagents, Italy. MgCl_2_ and CaCl_2_ solutions were freshly prepared in deionized water at concentrations of 30% *w*/*v* and 35% *w*/*v*, respectively, with similar concentrations reported previously [2,3]. Samples of Dead Sea solution were collected in 3 L containers a few days prior to the experiments (Figure 1). Samples were analyzed for Na, K, Ca, Mg and Sr by ICP-AES (Optima 3300, Perkin Elmer, Seer Green, UK) and Br by ICP-MS (NexION 300D, Perkin Elmer, Seer Green, UK). Cl concentrations were calculated by subtracting Br concentrations, analyzed by ICP-MS, from the total Br and Cl concentrations obtained using AgNO3 titration. Titrations were performed using an 848 Titrino plus (Metrohm, Herisau, Switzerland). Chemical analysis was performed at the Geological Survey of Israel. The chemical composition of the Dead Sea salt solutions is presented in Table 1.

#### 2.2.2. Application of Stabilizers

Soils were placed in trays customized to fit the wind tunnel dimensions (surface area of 0.5 m × 1.0 m and height of 0.02 m) (Figure 1). Brine solutions were applied to the soils by spraying the soil using a sprayer at equal volume to surface area ratios (1.5 L m^−2^). As controls, soils were either untreated or sprayed with tap water (clean drinking water). After applying the solutions and prior to the wind-tunnel experiments, the trays were left in the laboratory in order to avoid any environmental effect on the soils (e.g., wind-induced resuspension). Table 2 summarize the stabilization experimental matrix.

### 2.3. Boundary-Layer Wind Tunnel Experiments: Resuspension Testing and Calculations

Resuspension testing was performed at the Aeolian Simulation Laboratory, Ben-Gurion University of the Negev, using a boundary-layer wind tunnel [13]. Untreated and treated soils were tested following either 2 weeks or 6 weeks beginning from the day of treatment. The different times were chosen to represent different periods of aging following an incident. Experiments were conducted under four wind velocities, 5.3, 6.8, 8.1, and 9.6 m s^−1^, representing typical natural winds associated with dust emission in this region. ${PM}_{10}$ dust concentrations were recorded by light-scattering laser photometers DustTrak DRX 8534 (TSI Inc., Shoreview, MN, USA) placed 25 cm above the tunnel bed. Before placing the soil trays in the wind tunnel, ${PM}_{10}$ background levels of up to 20 μg m^−3^ were recorded. Background levels were subtracted from the ${PM}_{10}$ measurements, which were taken at different wind velocities. Each sample was measured for a duration of 30 s, at 1 s intervals. This short duration is enough to determine the dust emission patterns in controlled experiments [3,5]. Mass flux values of ${PM}_{10}$ resuspended from the ground (g m^−2^ s^−1^), expressed as $F\left({PM}_{10}\right)$, were calculated according to the following [13]:

(1) $$F\left({PM}_{10}\right)=\frac{C\left({PM}_{10}\right)\;\times \;Vt}{Ap\;\times \;t}$$

where $C\left({PM}_{10}\right)$ is the recorded ${PM}_{10}$ concentration (μg m^−3^), Vt is the air volume in the wind tunnel (3.43 m^3^), Ap is the area of the experimental plot (0.25 m^2^) and t is time (in seconds).

Mean mass flux values of ${PM}_{10}\;(\overset{-}{F}\left({PM}_{10}\right))$ were calculated by averaging all ${FPM}_{10}$ results per sample, i.e., 30 calculated flux values obtained over 30 s per wind velocity.

Saltating particles associated with the initiation of the dust emission process from soils [4,5] were collected by traps placed 2.5 to 10.5 cm above the tunnel bed and along the wind direction. Collected particles were weighted at the end of each experiment. Mean mass flux values of saltating particles (g m^−2^ s^−1^), expressed as $\overset{-}{F}(\text{saltation})$, were calculated according to the following:

(2) $$\overset{-}{F}(\text{saltation})=\frac{m(\text{saltation})}{Ap\;\times \;t}$$

where m(saltation) is the measured weight of the saltating particles (g), At is the cross-sectional area of the traps (0.02 m^−2^) and t is time (in seconds).

Suppression efficiencies (SE) of ${PM}_{10}$ or saltating particles (in percentage) were calculated for each stabilizer and soil type at each wind velocity according to the following:

(3) $$SE=\left(1-\frac{\overset{-}{F}}{\overset{-}{F}(\text{control})}\right)\times 100$$

where $\overset{-}{F}$ is the mean mass flux values of ${PM}_{10}$ or saltating particles (see above) and $\overset{-}{F}(\text{control})$ is the mean flux of the control sample (untreated) for the same wind velocity and soil type.

## 3. Results

### 3.1. Physicochemical Characteristics of the Soils

**Figure 2.**
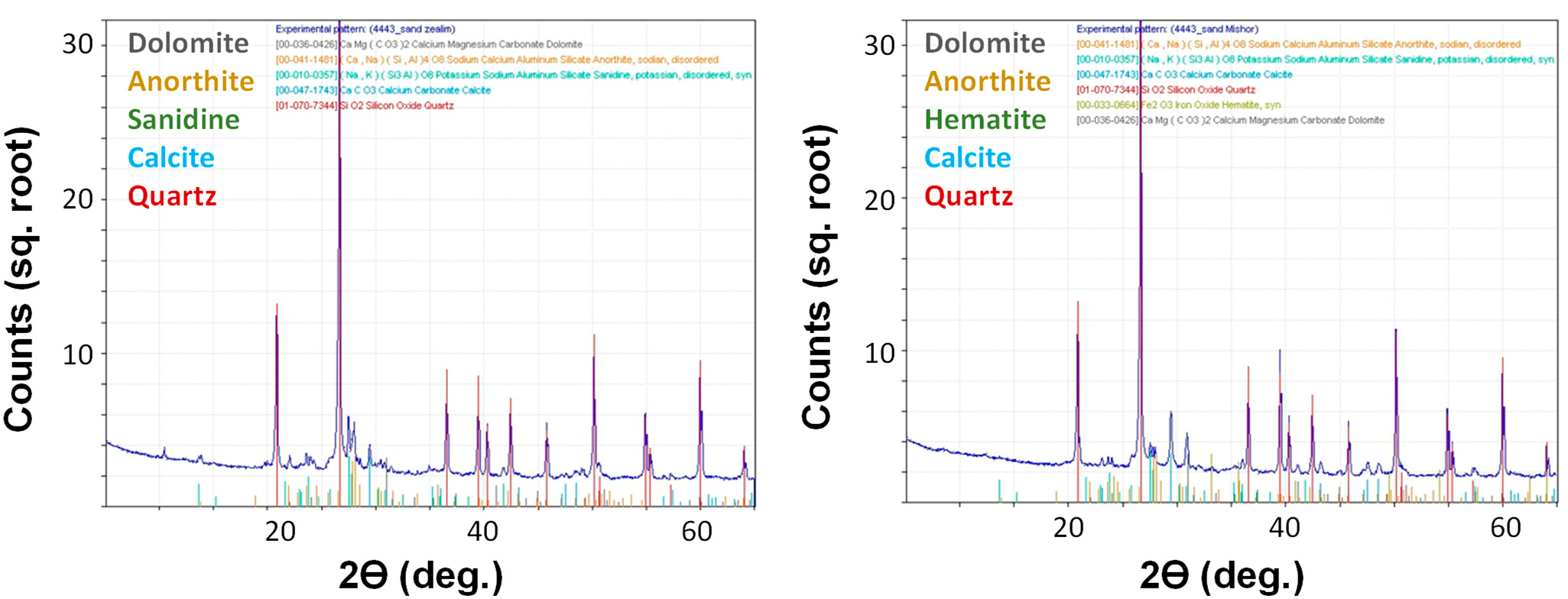
X-ray diffraction (XRD) patterns of the Ze’elim (**left** side) and Yamin soils (**right** side).

Soils were collected from two sites. The first sampling site was the Yamin plateau at the northern Negev Desert in Israel, and the second site was the Ze’elim sandy area at the western Negev Desert in Israel. Both soils are mainly composed of quartz (SiO_2_), silicate minerals (anorthite (CaAl_2_Si_2_O_8_), sanidine (CaAl_2_Si_2_O_8_)), carbonate minerals (dolomite (CaMg(CO_3_)_2_) and calcite (CaCO_3_)) and clay-sized minerals (hematite (Fe_2_O_3_)), as characterized by XRF and XRD analyses (Table 3, Figure 2). Additional analysis showed the soils were alkaline and contained low water and organic matter contents (Table 4), which are typical characteristics of desert soils.

**Figure 3.**
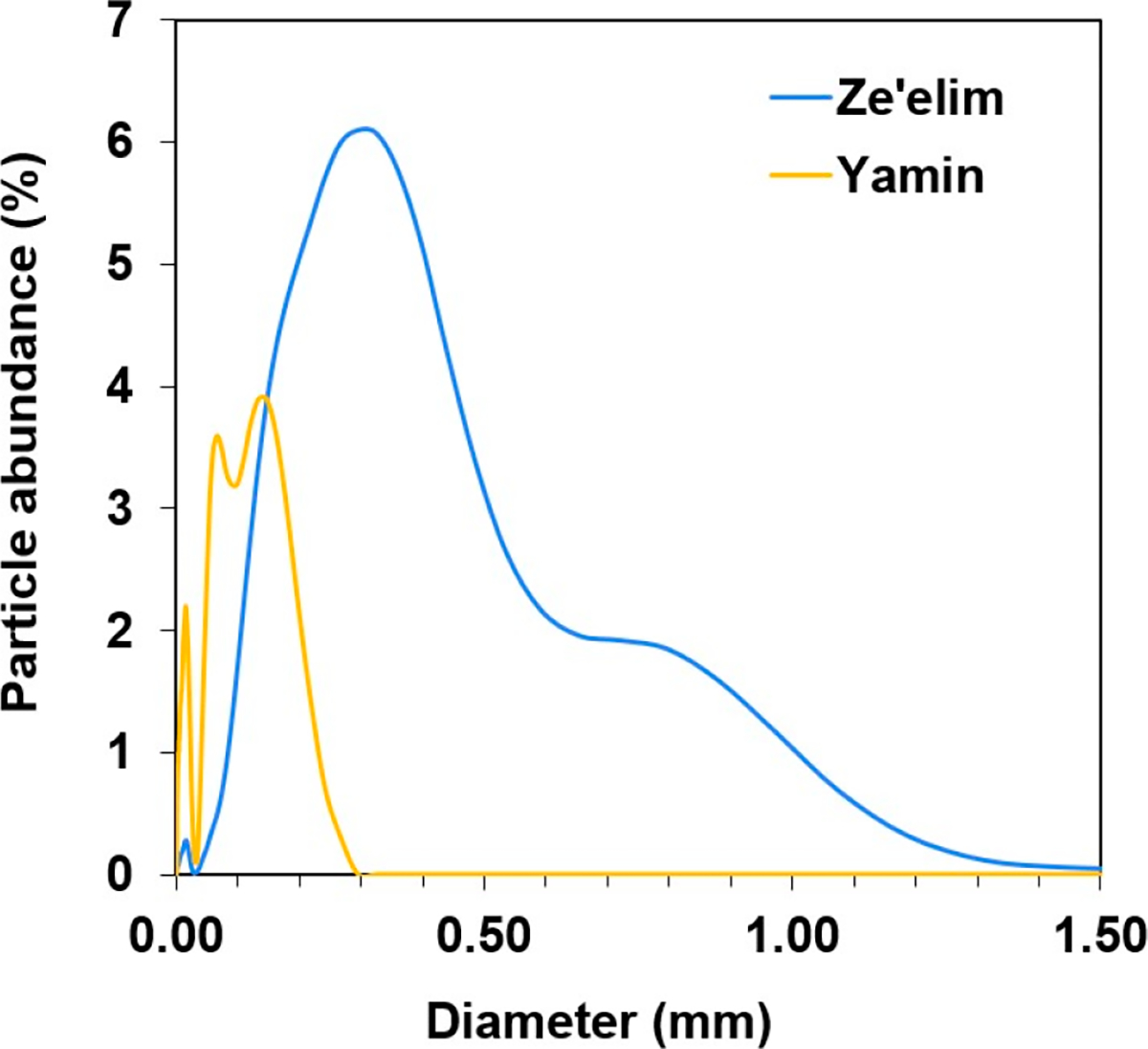
Particle size distribution of the Ze’elim and the Yamin soils in Israel.

PSD analysis showed different characteristics in grain size, whereas the Ze’elim soil was classified as sand, the Yamin soil was classified as silt loam (Figure 3). The Ze’elim soil demonstrated a higher mean grain size (170 μm vs. 50 μm) and a lower ${PM}_{10}$ content (3% vs. 28%) than the Yamin soil (Table 5). It was found that the Ze’elim soil is mainly composed of fine and medium sand fractions, while silt and fine sand are the main fractions in the Yamin soil.

### 3.2. Effectiveness of Brine Stabilizers on Resuspension Suppression from the Ze’elim Soil

To test the impact of the brine stabilizers on the resuspension from the soils, soils were treated with different stabilizers, left to dry for two weeks, and then tested for wind-induced dust emission. Untreated soils served as non-stabilized controls (NSCs). Soils were treated with either of the following stabilizers: CaCl_2_, MgCl_2_ and saltwater from the Dead Sea in Israel. Soils were also treated with tap water in order to control for the impact of wetting (Table 2).

**Figure 4.**
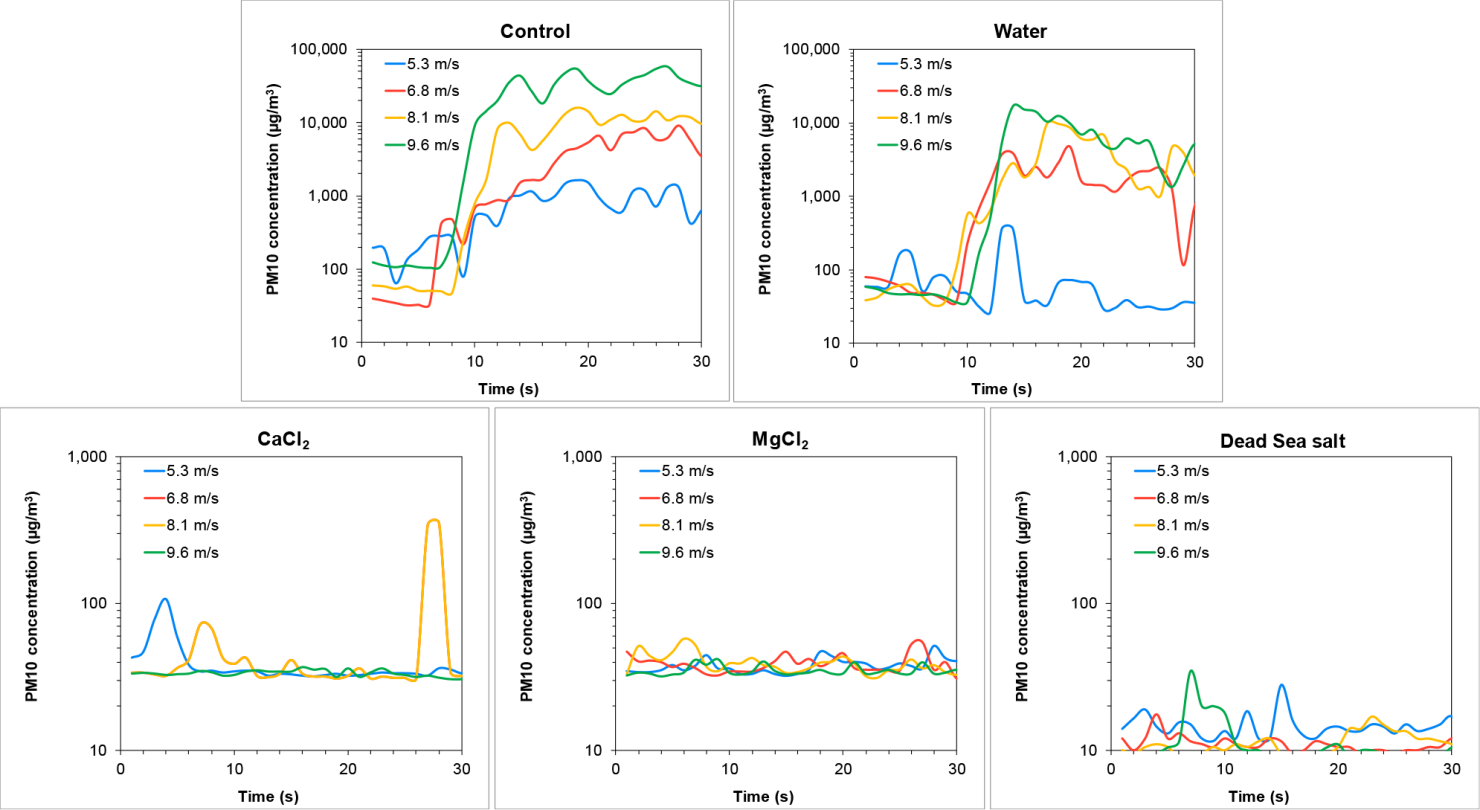
Wind-driven ${PM}_{10}$ emissions from the Ze’elim soil two weeks following treatment with stabilizers. Note the differences in the values of the Y-axis between Control and Water to the brines.

${PM}_{10}$ concentrations recorded during the wind tunnel experiment, representing wind-induced dust emissions from the Ze’elim soil, are presented in Figure 4. Higher wind velocities resulted in higher ${PM}_{10}$ resuspension levels from the untreated soil (control).

Resuspension was slightly reduced from soils sprayed with tap water (followed by drying) at all wind velocities tested, with a significant reduction at the lowest wind velocity. Extremely low resuspension levels were detected in brine treated soils, demonstrating that the soils were effectively stabilized following the treatments. The most effective dust suppressor was the Dead Sea salt treatment, yielding average ${PM}_{10}$ concentrations similar to background levels (~20 μg/m^3^).

**Figure 5.**
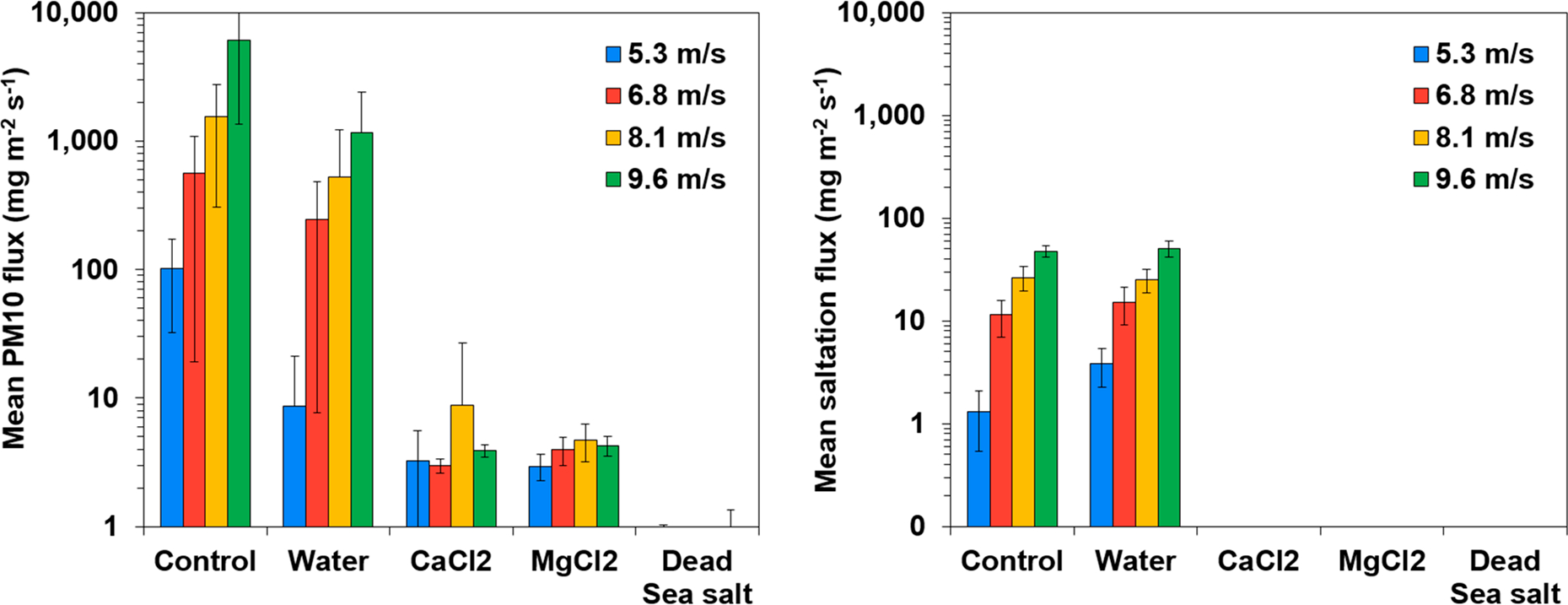
Wind-driven ${PM}_{10}$ and saltation fluxes from the Ze’elim soil treated with stabilizers.

Based on the ${PM}_{10}$ concentrations recorded during the wind tunnel experiment and the mass measurements of the collected salting particles, mean ${PM}_{10}$ fluxes and mean saltation fluxes were calculated, respectively. Figure 5 show the mean ${PM}_{10}$ fluxes and mean saltation fluxes from the Ze’elim soil under different treatment conditions, tested under four wind velocities. From these results, it was evident that the resuspension fluxes of saltating particles were significantly lower (by at least an order of magnitude) than dust particles, supporting that ${PM}_{10}$ are the major resuspension contributors under natural conditions.

To quantitatively evaluate the impact of the treatments on the resuspension of ${PM}_{10}$ and saltating particles, suppression efficiencies were calculated (Tables 6 and 7). Treating the Ze’elim soil with brine solutions resulted in effective stabilization, as shown by significantly reduced fluxes compared to the control and high resuspension suppression efficiencies of >97% (Figure 5, Tables 6 and 7) for all experimental conditions. The impact of the brine solutions was directly related to the salt treatment, as slightly reduced ${PM}_{10}$ fluxes and unchanged saltation fluxes were observed in soils misted with tap water only.

While all salt solutions efficiencies may be operationally relevant, interestingly, the most effective suppression effect on overall resuspension was achieved by the Dead Sea salt treatment, yielding 100% suppression efficiency over all wind velocities tested. For the prepared calcium and magnesium salt solutions, the efficiencies were less for lower wind speeds.

**Figure 6.**
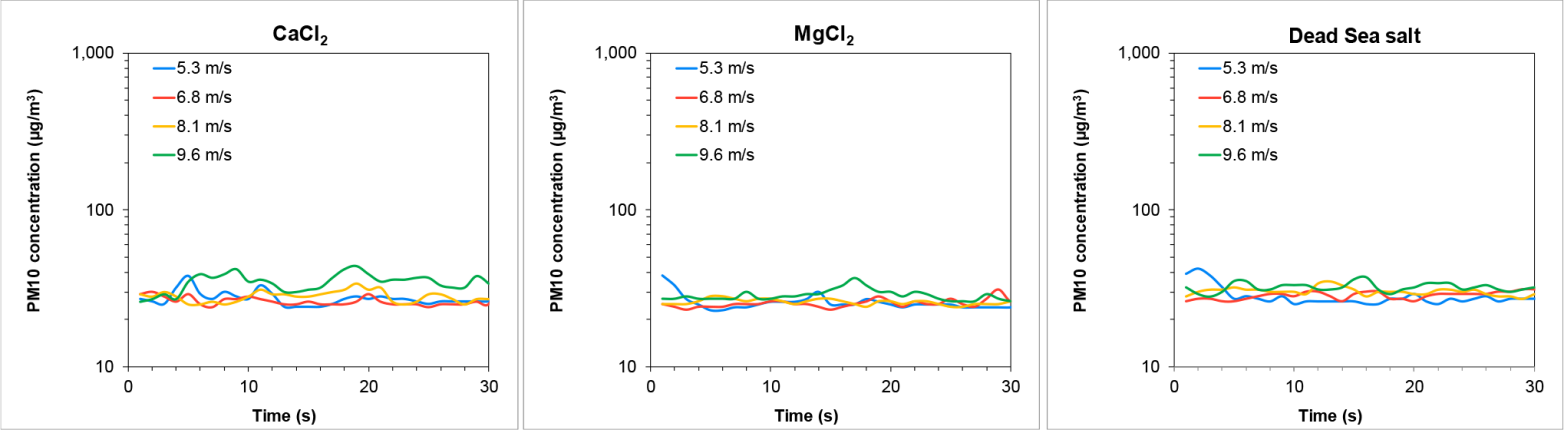
Wind-driven ${PM}_{10}$ emissions from the Ze’elim soil six weeks following treatment with stabilizers.

To evaluate the durability of the stabilization technique, re-testing was performed four weeks following the wind tunnel experiments described above (six weeks from the day of treatment). These time points were chosen because while operations may start immediately, they may continue over several weeks, so it is necessary to study the longer-term effectiveness. Re-testing resuspension of ${PM}_{10}$ concentrations from the Ze’elim soil is presented in Figure 6. Treatment with all three stabilizers resulted in low average ${PM}_{10}$ concentrations similar to background levels (~20 μg/m^3^). Resuspension levels of saltating particles were undetected (no particles were collected). These results demonstrated that treating the Ze’elim soil with brine solutions resulted in effective stabilization six weeks post-treatment.

### 3.3. Effectiveness of Brine Stabilizers on Resuspension Suppression from the Yamin Soil

**Figure 7.**
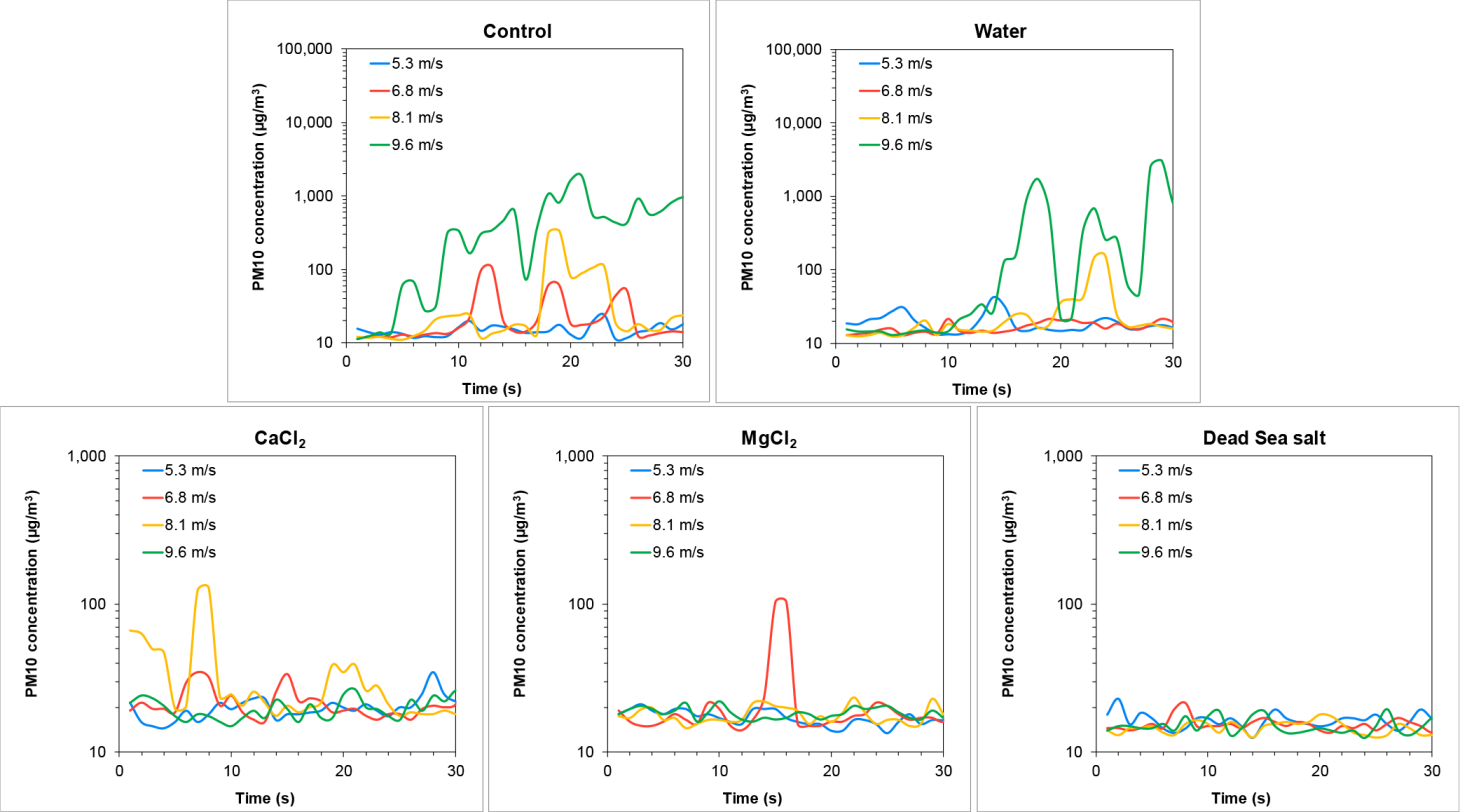
Wind-driven ${PM}_{10}$ emissions from the Yamin soil two weeks following treatment with stabilizers. Note the differences in the values of the Y-axis between Control and Water to the brines.

Yamin soil was subjected to treatments and resuspension testing similar to those performed on the Ze’elim soil. Soils were treated with different stabilizers, left to dry for two weeks, and then tested for particle emission in the wind tunnel. ${PM}_{10}$ concentrations recorded during the experiment, representing wind-induced dust emission from the Yamin soil following different treatments, are presented in Figure 7.

As shown for the Ze’elim soil, higher wind velocities resulted in higher ${PM}_{10}$ resuspension levels from the untreated Yamin soil (control). In contrast, significantly lower (by at least an order of magnitude) resuspension levels were observed from this soil when compared with the Ze’elim soil, as demonstrated by lower ${PM}_{10}$ concentrations recorded under identical conditions (Figures 4 and 7, control).

As shown in Figure 7, extremely low resuspension levels were detected in brine treated soils, demonstrating that the soils were effectively stabilized following the treatments. The Dead Sea salt treatment was the most effective dust suppressor for the Yamin soil, similar to the results obtained for the Ze’elim soil.

**Figure 8.**
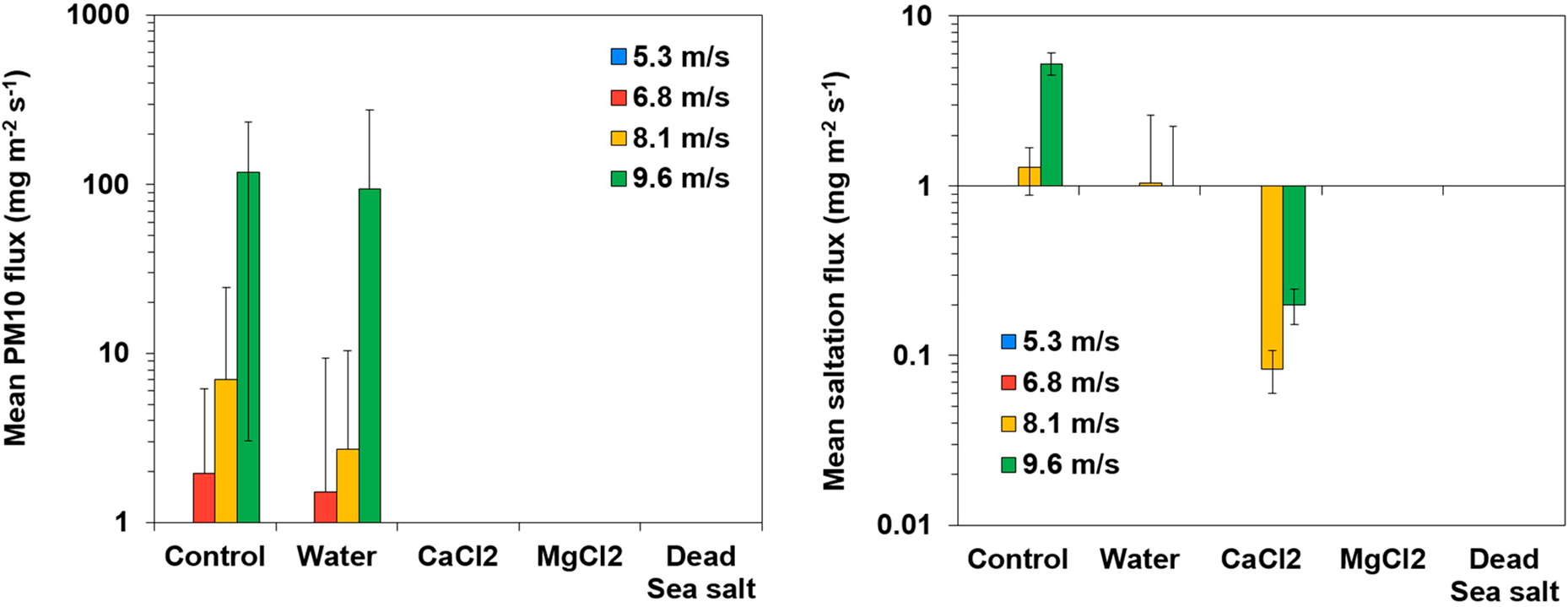
Wind-driven ${PM}_{10}$ and saltation fluxes from the Yamin soil treated with stabilizers.

Figure 8 show the mean ${PM}_{10}$ fluxes and the mean saltation fluxes from the Yamin soil under different treatments, tested under four wind velocities. As shown for the Ze’elim soil, the resuspension fluxes of saltating particles from the Yamin soil were significantly lower (by at least an order of magnitude) than dust particles, supporting that ${PM}_{10}$ are the major resuspension contributors under untreated conditions.

Tables 8 and 9 present the calculated suppression efficiencies of ${PM}_{10}$ and saltating particle resuspension from the Yamin soils. Suppression efficiencies could not be calculated under the lowest wind velocity because ${PM}_{10}$ measurements were low (around background levels), and no saltating particles could be collected and measured (noted NA). Treating the soil with brine solutions resulted in effective stabilization, as shown by significantly reduced fluxes compared to the control, along with resuspension suppression efficiencies (>94%). The most effective suppression effects on overall resuspension were achieved by the MgCl_2_ and Dead Sea salt treatments, yielding 100% suppression efficiency over all wind velocities tested.

**Figure 9.**
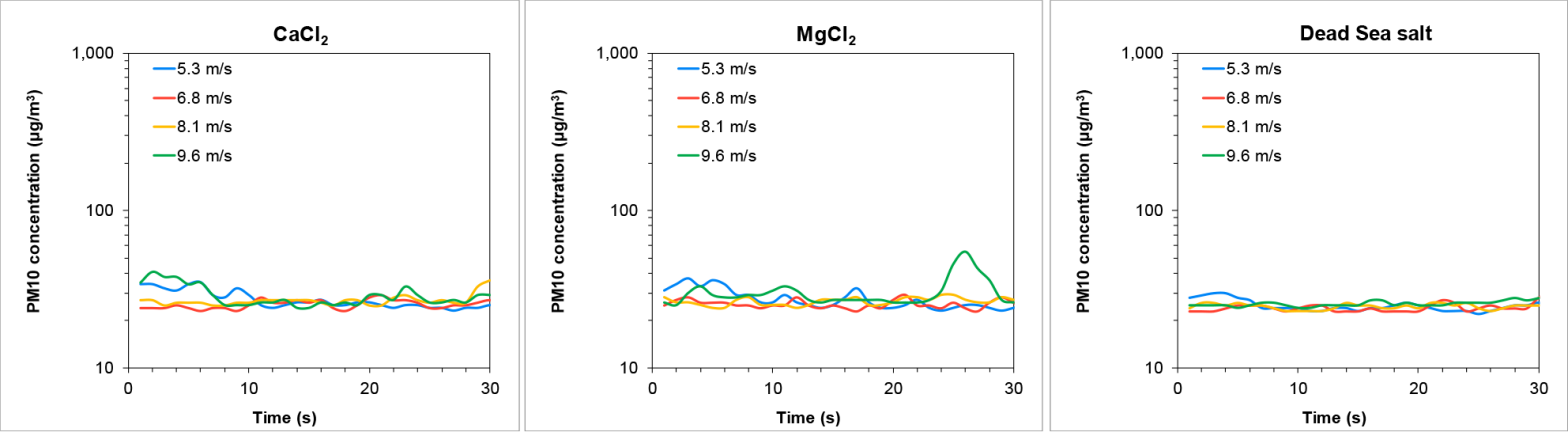
Wind-driven ${PM}_{10}$ emissions from the Yamin soil six weeks following treatment with stabilizers.

Analogous to the Ze’elim soil, the durability of the stabilization technique was evaluated on the Yamin soil by retesting resuspension from the treated trays following four additional weeks. Figure 9 present the ${PM}_{10}$ concentration recorded during the wind tunnel experiment from the Yamin soil. Treatment with all the three stabilizers resulting in average ${PM}_{10}$ concentrations similar to background levels (~20 μg/m^3^). Resuspension levels of saltating particles were undetected (no particles were collected). These result demonstrated that treating the Yamin soil with brine solutions resulted in effective stabilization even six weeks post-treatment.

## 4. Discussion

Treating the two soils with salt/brine solutions resulted in reduced particle resuspension, as shown by extremely low ${PM}_{10}$ fluxes (equivalent to background levels) and high resuspension suppression efficiencies (>94%). The impact of the brine solutions was directly related to the salt treatment rather than the wetting of the soils since similar particle resuspension fluxes were obtained from untreated soils or soils sprayed with tap water only. Brine solutions are, therefore, effective stabilizers, leading to reduced resuspension of soil particles. These results are consistent with previous work performed by Katra et al. [3], which tested the impact of diverse dust control products of synthetic and organic polymers (Lignin, Resin, Bitumen, PVA, Brine) on unpaved roads. The authors showed that some products significantly reduced dust emission from quarry roads, especially when using magnesium chloride (Brine).

All three salt\brine solutions tested in this study function primarily by helping cement small particles into larger ones that are more difficult to resuspend [2]. Their capability to enhance the cohesion of smaller particles is expected to vary with the composition of the salt solution, as well as the specific particles involved. Aiding in this cohesion is the fact that salts such as CaCl_2_ and MgCl_2_ are hygroscopic, so when they dry out after being applied (usually by spraying an aqueous solution), some water may be present, which helps enhance cohesion [2]. The effectiveness of the stabilizers is expected to occur immediately after the applied solutions dry, which in desert climates is expected to not take long, as shown in this work. While all salt solutions have operational relevance, the most effective stabilizer was the Dead Sea salt solution, yielding 100% resuspension suppression efficiency of ${PM}_{10}$ and saltating particles over all wind velocities tested. The motivation to test the Dead Sea salt solution as a stabilizer was it being an easily available, natural resource of salts. Saltwater from the Dead Sea can be derived directly from the sea or procured locally. MgCl_2_ and CaCl_2_ were also highly effective but slightly less effective than the Dead Sea salt in limiting ${PM}_{10}$ resuspension from the Ze’elim soil (>97%). CaCl_2_ was also slightly less effective in limiting the resuspension of saltating particles from the Yamin plateau (>94%). The Dead Sea solution is expected to contain other substances, such as specific ions and humic substances that help retain hydration, which may enhance the cohesion of small particles.

Significantly lower resuspension levels were observed from the Yamin soil when compared with the Ze’elim soil (>10-fold difference), indicated by lower ${PM}_{10}$ concentrations recorded under identical conditions (Figures 6 and 9). This may result from differences in the cohesiveness of the soil particles between the two soil types, rather than the content of the ${PM}_{10}$ in the soil (Table 5), which is significantly higher in the Yamin than the Ze’elim soil (28 wt% and 7 wt%, respectively). It demonstrates the role of sand transport in dust-${PM}_{10}$ emission from sandy soils [14].

Resuspension fluxes of saltating particles from the two soils were >10-fold lower than dust particles, demonstrating that ${PM}_{10}$ are the major resuspension contributors under natural conditions. This result confirms that dust emission is expected to cause the major spread of the contamination in the case of an emergency event in the Negev desert, highlighting the importance of limiting resuspension of contaminated dust. Treating the soils with brine solutions resulted in effective stabilization six weeks post-treatment, supporting that this technique can effectively limit resuspension of contaminated soil after an emergency event for a prolonged duration, allowing sufficient time for decision making and management of further actions. This is particularly important in desert environments where continued drying could otherwise lead to increased resuspension.

Our results highlight the importance of considering the soil properties at a specific site when considering the impacts and mitigation of resuspension. The two soils in this study have characteristics that contribute to their ability to be resuspended, e.g., small organic content and low moisture content. Therefore, they may be considered “worst cases”, such that the results may also be applicable to many other types of soils for which resuspension may be inherent less favored.

While the salt solutions appear to increase the cohesiveness of small particles and thus reduce wind-induced resuspension, complex mechanisms appear to govern the disintegration of the cohesive/cemented particles and their subsequent resuspension. Therefore, to validate the applicability of stabilization techniques, it is essential to test the impact of stabilizers in specific situations which induce different types of physical stresses other than wind. Two operationally relevant cases are the movement of vehicles and foot traffic. EPA investigated simulated vehicle and foot traffic in controlled laboratory studies [15]. Together, the results of the present study, along with the EPA study, suggest the relevancy and urgency of testing stabilization techniques on a larger scale area under natural environmental conditions.

## Figures and Tables

**Table 1.** Chemical composition of the stabilization solutions.

Ion/Element	Stabilization Material		
	CaCl_2_	MgCl_2_	Dead Sea Salt Water

Na^+^	-	-	8700
K^+^	-	-	13,600
Ca^++^	127,000	-	28,800
Mg^++^	-	76,600	74,000
Cl^−^	223,000	223,000	291,000
Br^−^	-	-	8400

**Table 2.** Stabilization experimental matrix.

Soil	Non-Stabilized Control (Untreated)	Tap Water	Stabilization Material		
			CaCl_2_	MgCl_2_	Dead Sea Salt

Yamin	A	B	C	C	C
Ze’elim	A	B	C	C	C

A Testing of untreated soils

B Testing of soils 2 weeks post-treatment

C Testing of soils 2 weeks and 6 weeks post-treatment.

**Table 3.** χ-ray fluorescence (XRF) measurements of soils from the Ze’elim area and the Yamin Plateau in Israel.

Compound	Ze’elim Soil (wt%)	Yamin Soil (wt%)

SiO_2_	89	84
Al_2_O_3_	5	3
CaO	2	8
Fe_2_O_3_	2	2
K_2_O	1	1
SiO_2_	89	84

**Table 4.** Soil properties of the Ze’elim and the Yamin soils in Israel.

Properties	Ze’elim Soil (wt%)	Yamin Soil (wt%)

pH	7.9	7.8
Water content (wt%)	<1	<1
Total organic content (wt%)	<0.3	<0.3

**Table 5.** Particle size fractions of the Ze’elim and the Yamin soils in Israel.

Size Fraction		Ze’elim Soil (%)	Yamin Soil (%)

Clay (<0.002 mm)		1	7

Silt (0.002–0.063 mm)		6	51

Sand	Fine (0.063–0.25 mm)	37	41
	Medium (0.25–0.5 mm)	39	1
	Coarse (0.5–2.0 mm)	77	0

PM_10_ (<0.01 mm)		3	28

**Table 6.** Suppression efficiencies of wind-driven ${PM}_{10}$ emission from the Ze’elim soil treated with stabilizers.

Wind Velocity/Treatment	5.3 m/s	6.8 m/s	8.1 m/s	9.6 m/s

Tap Water	92%	56%	66%	81%
CaCl_2_	97%	99%	99%	100%
MgCl_2_	97%	99%	100%	100%
Dead Sea salt	100%	100%	100%	100%

**Table 7.** Suppression efficiencies of wind-driven saltating particle emission from the Ze’elim soil treated with stabilizers.

Wind Velocity/Treatment	5.3 m/s	6.8 m/s	8.1 m/s	9.6 m/s

Tap Water	0%	0%	4%	0%
CaCl_2_	100%	100%	100%	100%
MgCl_2_	100%	100%	100%	100%
Dead Sea salt	100%	100%	100%	100%

**Table 8.** Suppression efficiencies of wind-driven ${PM}_{10}$ emission from the Yamin soil treated with stabilizers.

Wind Velocity/Treatment	5.3 m/s	6.8 m/s	8.1 m/s	9.6 m/s

Tap Water	NA ^1^	22%	61%	21%
CaCl_2_	NA ^1^	100%	100%	100%
MgCl_2_	NA ^1^	100%	100%	100%
Dead Sea salt	NA ^1^	100%	100%	100%

1 Not available (NA) means values could not be calculated because the mean mass of flux of the control sample was zero.

**Table 9.** Suppression efficiencies of wind-driven saltating particle emission from the Yamin soil treated with stabilizers.

Wind Velocity/Treatment	5.3 m/s	6.8 m/s	8.1 m/s	9.6 m/s

Tap Water	NA ^1^	NA ^1^	19%	81%
CaCl_2_	NA ^1^	NA ^1^	94%	96%
MgCl_2_	NA ^1^	NA ^1^	100%	100%
Dead Sea salt	NA ^1^	NA ^1^	100%	100%

1 Not available (NA) means values could not be calculated because the mean mass of flux of the control sample was zero.

## Data Availability

Data are contained within the article.
